# Reverse genetic characterization of the natural genomic deletion in SARS-Coronavirus strain Frankfurt-1 open reading frame 7b reveals an attenuating function of the 7b protein *in-vitro *and *in-vivo*

**DOI:** 10.1186/1743-422X-6-131

**Published:** 2009-08-24

**Authors:** Susanne Pfefferle, Verena Krähling, Vanessa Ditt, Klaus Grywna, Elke Mühlberger, Christian Drosten

**Affiliations:** 1 Clinical Virology Group, Bernhard Nocht Institute for Tropical Medicine, Hamburg, Germany; 2 Department of Virology, Philipps University Marburg, Germany; 3 Institute of Virology, University of Bonn Medical Centre, Bonn, Germany; 4 National Infectious Diseases Laboratories Institute, Boston, USA; 5 Department of Microbiology, Boston University School of Medicine, Boston, USA

## Abstract

During the outbreak of SARS in 2002/3, a prototype virus was isolated from a patient in Frankfurt/Germany (strain Frankfurt-1). As opposed to all other SARS-Coronavirus strains, Frankfurt-1 has a 45-nucleotide deletion in the transmembrane domain of its ORF 7b protein. When over-expressed in HEK 293 cells, the full-length protein but not the variant with the deletion caused interferon beta induction and cleavage of procaspase 3. To study the role of ORF 7b in the context of virus replication, we cloned a full genome cDNA copy of Frankfurt-1 in a bacterial artificial chromosome downstream of a T7 RNA polymerase promoter. Transfection of capped RNA transcribed from this construct yielded infectious virus that was indistinguishable from the original virus isolate. The presumed Frankfurt-1 ancestor with an intact ORF 7b was reconstructed. In CaCo-2 and HUH7 cells, but not in Vero cells, the variant carrying the ORF 7b deletion had a replicative advantage against the parental virus (4- and 6-fold increase of virus RNA in supernatant, respectively). This effect was neither associated with changes in the induction or secretion of type I interferon, nor with altered induction of apoptosis in cell culture. However, pretreatment of cells with interferon beta caused the deleted virus to replicate to higher titers than the parental strain (3.4-fold in Vero cells, 7.9-fold in CaCo-2 cells).

In Syrian Golden Hamsters inoculated intranasally with 10e4 plaque forming units of either virus, mean titers of infectious virus and viral RNA in the lungs after 24 h were increased 23- and 94.8-fold, respectively, with the deleted virus. This difference could explain earlier observations of enhanced virulence of Frankfurt-1 in Hamsters as compared to other SARS-Coronavirus reference strains and identifies the SARS-CoV 7b protein as an attenuating factor with the SARS-Coronavirus genome. Because attenuation was focused on the early phase of infection *in-vivo*, ORF 7b might have contributed to the delayed accumulation of virus in patients that was suggested to have limited the spread of the SARS epidemic.

## Introduction

The severe acute respiratory syndrome (SARS) emerged in the end of 2002 in China and caused an international epidemic [1]. Its causative agent, a hitherto unknown Coronavirus (CoV) is thought to have been circulating in an animal reservoir before it crossed species barriers into humans [2-7]. Bats have been implicated as the original reservoir of all CoV, and the large range of relevant human and animal CoV has been suggested to be resulting from host switching events [8-16].

In the context of viral host switching, it is interesting that several SARS-CoV proteins encoded on subgenomic (sg) RNAs seem to be dispensable for virus replication in cultured cells of primate or rodent origin, as well as in rodent models [17-19]. Because these ORFs are not shared between different CoV groups, they are referred to as group-specific ORFs [20]. Proteins encoded by group-specific ORFs have been shown to influence pathogenesis, virus replication, or host immune response [17,20-24]. During the human SARS epidemic, SARS-CoV has rapidly acquired deletions in several of its group-specific ORFs [7,25-27]. The original functions of associated proteins might exemplify mechanisms through which highly pathogenic zoonotic viruses such as the SARS-CoV can persist in their reservoirs without causing disease.

The characterization of virus proteins can be unreliable if only the protein of interest is studied on its own. The study of proteins in the whole virus context reflects virus-host interactions more realistically, and takes into account intraviral protein interactions. Such experiments can be done using reverse genetics techniques which for most plus-strand viruses rely on cloned cDNA copies of the whole RNA genome that can be mutagenized *in-vitro *[28-30]. Different approaches have been followed to implement CoV reverse genetics. A great challenge in this regard is the huge size of the CoV genome, making cloning procedures difficult because plasmid-based cDNA constructs are instable in *E. coli*. *In-vitro *ligation of subgenomic cDNA fragments without the assembly of full-length plasmids has been successfully used to establish CoV reverse genetics [31-33]. As an alternative, full-length cDNA copies have been reconstructed and kept in vaccinia virus [34,35]. A third approach is based on bacterial artificial chromosomes (BAC) for keeping full-length CoV cDNA stable, owing to a low copy number of BAC DNA per *E. coli *cell [36-39]. The first two systems use T7 RNA polymerase promoter-driven *in-vitro *transcription of capped, infectious RNA that is transfected into cells. The latter uses a CMV promoter and relies on the transfection of full-length cDNA into cells, which is then transcribed in the nucleus into infectious RNA. In this study we have implemented a modified approach to CoV reverse genetics by cloning the entire SARS-CoV genome downstream of a T7 RNA polymerase promotor in a BAC. Using the linearized BAC construct as a template for *in vitro *transcription, this system combines plasmid-based handling of cDNA constructs with direct delivery of genome-like RNA into the cytoplasm.

The novel system was used to characterize a 45 nucleotide in-frame deletion in ORF 7b that is present in the primary isolate of SARS-CoV prototype strain Frankfurt-1 [20]. This specific deletion is not present in any other of > 150 SARS-CoV ORF 7b sequences in GenBank, and in none of the SARS-like bat CoV. However, deletions of the whole ORF 7b and beyond have been acquired by SARS-CoV during the SARS epidemic in humans [25-27].

The ORF 7b protein is a 44 amino acid protein that is transcribed by a noncanonical leaky scanning mechanism from the second ORF encoded on subgenomic RNA 7 [20,40]. The protein is a type III integral transmembrane protein located in the Golgi compartment [41]. It has been shown previously that the protein is a structural virion component, that it is dispensable for replication in various cell cultures, and that it induces apoptosis in cultured cells if overexpressed [18,40]. The pro-apoptotic effect seems to be limited to late stages of the apoptotic cascade [18]. Qualitatively the same effect was confirmed in studies on a recombinant virus, containing a combined deletion of ORF 7a and ORF 7b [18]. However, it is unclear to what extent either the ORF 7a or ORF 7b proteins, respectively, contribute to the effect. It is also unclear to what degree the ORF 7b protein alone influences virus replication *in-vivo*. This is relevant for the Frankfurt-1 virus because it has been used as a model virus in several studies on pathogenesis and antiviral drug research (e.g [42-45]). Finally it is unclear whether the Frankfurt-1 ORF 7b deletion has been acquired during cell culture, or whether it may have been present already in the patient and may have undergone transmission.

In this study, primary clinical samples from the Frankfurt index patient and a secondary case who acquired her infection from him were re-analyzed. Frankfurt-1 viruses with and without the deletion were then reconstructed by reverse genetics. Effects of the deletion on interferon induction and response, on induction of apoptosis, and on *in-vivo *replication in Syrian Golden hamsters were determined.

## Results

### Origin of the ORF 7b deletion

**Figure 1:**
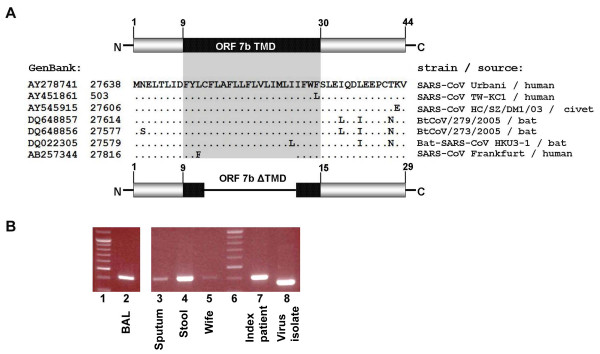
**Amino acid variability in ORF 7b and RT-PCR analysis of ORF 7b in clinical samples versus cell culture isolate**. (A) ORF 7b amino acid alignment of all SARS- and SARS-like CoV available in GenBank (sequences yielding identical alignments in the region of interest were deleted). The transmembrane domain [41] is shaded in black/grey. The left column shows GenBank accession numbers of representative genomes for each unique amino acid sequence, along with the starting nucleotide positions of ORF 7b in each GenBank entry. The right hand column shows strain designations and their sources (human, civet, bat). Only one sequence derived from the Frankfurt-1 strain (AB257344) shows a 45 nucleotide in-frame deletion in the predicted transmembrane domain (TMD). The drawing below the alignment panel represents the ORF 7b in recombinant virus r7bΔTMD. (B) Amplification of a 403 bp fragment of ORF 7b by RT-PCR in clinical samples taken after the initial isolation of strain Frankfurt-1 from the Frankfurt index patient (bronchoalveolar lavage sample (BAL) [lane 2], sputum sample [lane 3] stool sample [lane 4]), as well as a sputum sample from the wife of the index patient (wife, lane 5) [2]. Lane 7 shows the corresponding amplification product in the original sputum sample that yielded the Frankfurt-1 isolate. Lane 8 depicts the PCR product of the virus isolate derived from this sample.

The Frankfurt-1 SARS-CoV cell culture isolate contained a 45 nt in-frame deletion within a predicted transmembrane region. A back-translated BLAST search on the nucleotide database (tBLASTn) showed that this deletion was not present in any of > 150 SARS-CoV ORF 7b sequences in GenBank (except in an independent sequence of the Frankfurt strain), and in none of 8 SARS-like bat-CoV sequenced in the ORF 7b region (Figure 1). To determine whether the deletion originated from the infected patient or was generated in cell culture, RT-PCR was used to screen for the deletion in several sequential samples from the Frankfurt index patient of whom the Frankfurt-1 isolate had been taken. As shown in Figure 1, all patient samples yielded DNA bands of higher molecular weight than those from the cell culture isolate, indicating absence of the deletion in the patient. Of note, clinical samples from the wife of the index patient, who got infected by her husband in later course, did not contain the deletion either (Figure 1). To exclude that a minor background of the virus population in patient samples might have carried the deletion already prior to virus isolation, a second PCR was done with a primer bridging the deleted region (i.e., it bound up- and downstream of the deletion and would only amplify deletion-containing viruses). The deleted virus could not be detected in any patient sample. It was therefore assumed that the virus had acquired the deletion during isolation in cell culture.

### Expression of ORF 7b but not ORF 7b with the 45 nt deletion induces apoptosis and the type I interferon response

**Figure 2:**
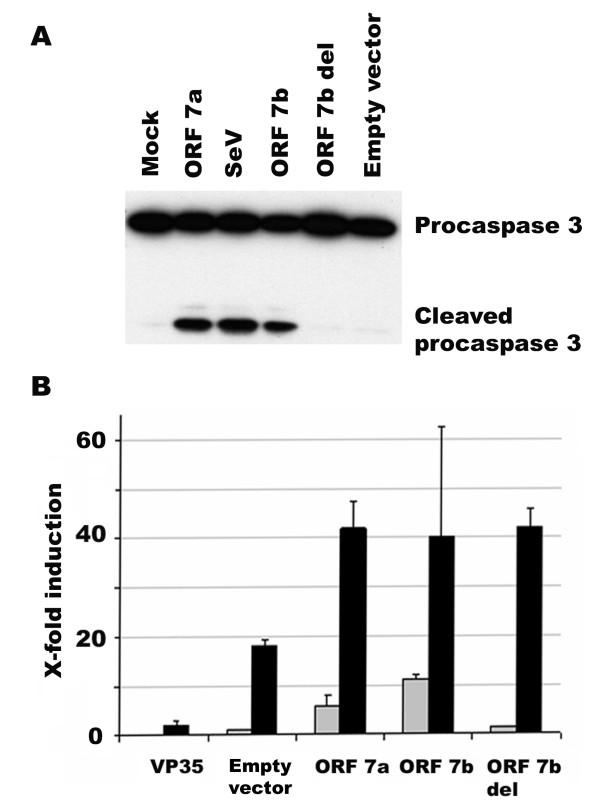
**Influence on apoptosis and type I interferon induction by overexpression of ORF 7a, ORF 7b, and ORF 7b with the Frankfurt-1-specific deletion**. (A) Cleavage of procaspase 3 analyzed by Western blot on cell lysates 48 h after transfection with indicated plasmids or infection with Sendai virus (20 hemagglutinating units). (B) Interferon beta promoter-specific reporter gene expression (y-axis), shown as the factor of induction as compared to the mock-transfected, non-superinfected control (see below). The assay was done by transfection of HEK 293 cells with plasmids expressing either Ebolavirus VP35, ORF 7a, ORF 7b, or ORF 7b with a deletion corresponding to the ORF 7b deletion in Frankfurt-1 (x-axis), as well as reporter constructs for the interferon beta promoter (Firefly luciferase) and the SV40 promoter (*Renilla *luciferase). 24 h post transfection, cells were either superinfected with SeV (20 hemagglutinating units) or left uninfected. Interferon-specific reporter gene expression was determined 24 h after superinfection (black bars) or mock infection (grey bars). The experiment was done in triplicate and standard deviations are shown. To determine interferon-specific expression, the Firefly luminescence signal was divided by the *Renilla *luciferase signal.

Several SARS-CoV accessory gene products have been shown to be involved in the induction of apoptosis, including the 7a and 7b proteins [18,46,47]. To analyze whether the deletion in ORF 7b had any influence on its ability to induce apoptosis, Vero E6 cells were transfected with expression plasmids encoding ORF 7a, ORF 7b or ORF 7b del containing a deletion exactly corresponding to that in Frankfurt-1. Control cells were infected with Sendai virus (SeV) or left untreated. Forty-eight hours later, lysates were analyzed for procaspase 3 cleavage by Western blot using an antibody that detects cleaved and non-cleaved forms. As shown in Figure 2A, cleavage of caspase 3 was observed in cells expressing ORF 7a and ORF 7b. Interestingly, expression of ORF 7b del did not cause caspase 3 cleavage.

To examine the effect of the ORF 7b deletion on the type I IFN response, reporter gene assays were performed. Cells were transfected with plasmids encoding ORF 7a, ORF 7b or ORF 7b del, respectively. All cells were co-transfected with the pHISG-54 reporter plasmid containing the firefly luciferase gene under the control of the ISRE region of the human IFN-stimulated gene 54. Expression plasmid pRL-SV40 encoding *Renilla *luciferase was co-transfected to normalize for interferon-independent stimulation of transcription. Twenty-four hours later, the cells were infected with SeV to induce IFN-mediated reporter gene expression. Cells were lyzed 24 h post infection and subjected to reporter gene assays. As shown in Figure 2B, expression of both ORF 7a and ORF 7b but not ORF 7b del induced IFN-dependent reporter gene expression. In those cultures superinfected with SeV, none of the plasmids reduced the SeV-associated, IFN-dependent reporter gene expression. The Ebolavirus VP35, a known antagonist of interferon induction, clearly showed reduction of reporter gene expression if used in the same system (Figure 2B) [48,49].

These distinct findings prompted us to elucidate 7b protein functions in the natural virus context. To be able to measure even marginal phenotypical differences we decided to reconstruct both genotypes while establishing a novel reverse genetic system.

### Construction of a full-length infectious cDNA clone

**Figure 3:**
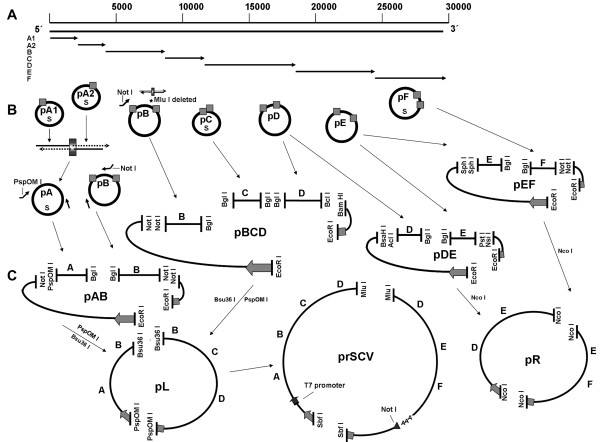
**Assembly of a full-length SARS-CoV cDNA clone in a BAC (refer to Materials and Methods section for a detailed description of construction steps)**. (A) Arrows symbolize positions of PCR fragments on the SARS-CoV genome. These were cloned in subgenomic plasmids. (B) Subgenomic plasmids pA1 - pF. Plasmids are either based on pSMART (identified by an "S" symbol within the respective clones) or on pCR2.1 (no symbol). Squares on each plasmid symbolize the approximate positions of erroneous mutations from initial cloning corrected by fragment-extension technique before assembly to higher-order clones. Small extension-PCR symbols above clones pB and pF indicate mutations introduced into plasmids to facilitate subsequent construction steps (deletion of an *Mlu *I-site in pB) or to fill in the 45 nt deletion in ORF 7b in pF. (C) Assembly of quarter clones. Circles represent plasmids, ovals represent BACs. Bold grey arrows symbolize essential BAC-encoded genes reconstituted during BAC ligation, in order to achieve high cloning efficiency. Restriction digestion steps are symbolized by thin arrows. The utilized restriction enzymes are identified next to the arrows. PCR primer symbols (small arrows) next to plasmids indicate that these plasmids were first amplified with primers introducing restriction sites (identified next to primer symbols) before the resulting products were double-digested as indicated. The large horizontal arrows below plasmids pA1 and pA2 indicate that these fragments were joined by overlap-extension PCR with primers eliminating a *Bgl *I restriction site as symbolized by a square on both of the parental plasmids. In each construction, fragment ends shown in close proximity were first ligated *in-vitro*. The ligation products were then purified, ligated at sites drawn in greater distance, and transformed in *E. coli*.

**Figure 4:**
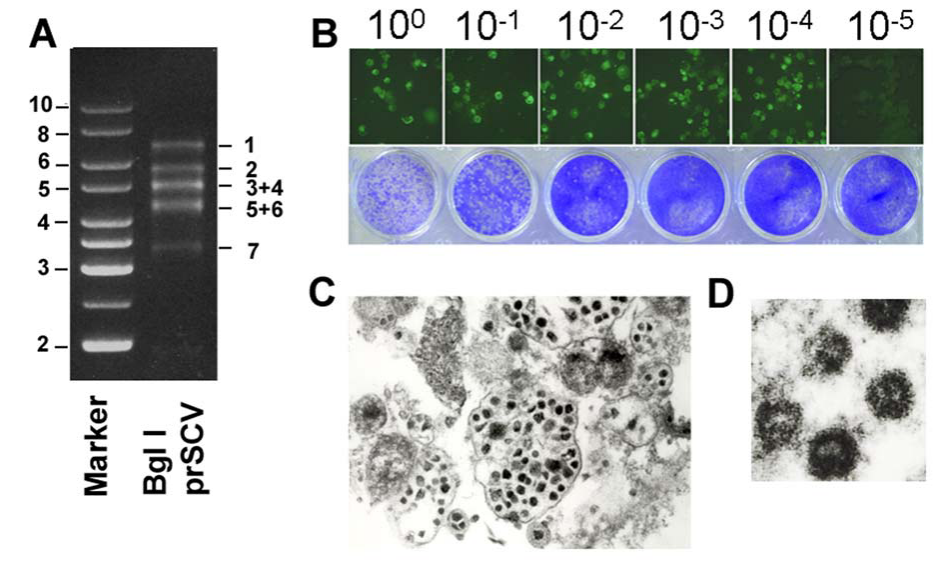
**Recovery of recombinant virus**. (A) Digestion of full-length BAC cDNA clone prSCV with the restriction enzyme *Bgl *I. The BAC construct had seven *Bgl *I restriction sites at positions 4454, 8783, 12146, 19000, 24124, 31719, and 36168, resulting in 7 digestion fragments as annotated in the gel picture: 7595 bp (infectious clone Fragment F as identified in Figure 3A with appending BAC fragment [digestion fragment 1]); 6854 bp (Fragment D, [2]); 5124 bp (Fragement E, [3]); 4972 bp (Fragment A with appending BAC fragment, [4]); 4449 bp (BAC fragment, [5]); 3362 bp (Fragment C, [6]); 4330 bp (Fragment B, [7]) (B) Analysis of supernatants taken from BHK cells 24 h after transfection with *in-vitro *transcripts from the BAC cDNA clone. Supernatant was diluted as indicated and plated on Vero cells. The top panel shows the results of indirect immunofluorescence analysis using a human polyclonal antiserum. The bottom panel shows the results of plaque assays on the same Vero cells. (C) Electron micrograph of Vero cells infected as described above. (D) Detail from (C).

In order to clone subgenomic portions of the SARS-CoV genome, seven PCR fragments covering the whole genome were generated with primers described by Yount *et al*. [32]. Fragments were initially cloned in high copy number plasmid vectors, or, if refractory to cloning, in low copy plasmids as shown in Figure 3. Except some marker mutations (see below), the sequence of cDNA inserts in the seven resulting subclones was corrected to match that of the cell culture-derived virus by plasmid-based inverse PCR and fragment-extension PCR. For construction of the variant with an intact ORF 7b, the 45 nt deletion was filled in by oligonucleotide extension PCR on subclone pF (Figure 3). Corrected subclones were assembled in a stepwise procedure into four BAC clones containing about a quarter of the SARS-CoV genome each, which where then joined into a full length BAC cDNA clone (refer to Figure 3 and the Materials and Methods section for more details on the construction). BACs containing both versions of subclone F were assembled. Both BACs were sequenced, confirming presence of all marker mutations and absence of any further mutations (refer to GenBank accession number FJ429166). One whole BAC was digested with Bgl I, which was present at seven positions on the BAC construct. As shown in Figure 4A, fragments of the expected sizes were obtained.

The linearized BAC cDNA and a PCR product containing the nucleocapsid gene were *in-vitro *transcribed and co-transfected in BHK cells. Because BHK cells did not support SARS-CoV replication, supernatants were transferred to Vero cells susceptible for SARS-CoV infection. Virus progeny was identified by immunofluorescence analysis with anti-SARS-CoV patient serum after 24 h (Figure 4B), as well as by plaque assays after 48 h (Figure 4B). Electron microscopy showed intracellular structures compatible with sites of virion assembly as well as mature virus particles (Figure 4C).

**Figure 5:**
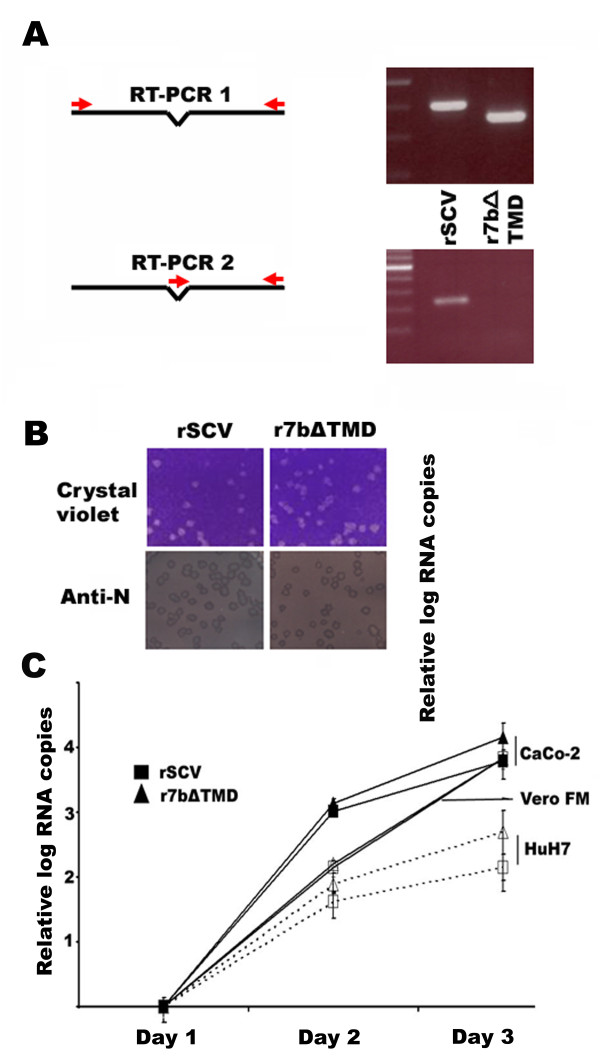
**Comparison of recombinant viruses rSCV and r7bΔTMD**. (A) RT-PCRs on supernatants of Vero cells spanning the region of the ORF 7b deletion (RT-PCR 1) or targeting the sequence deleted in ORF7bΔTMD (RT-PCR 2). rSCV is the full-length ORF 7b virus; r7bΔTMD is the virus with the Frankfurt-1-specific deletion in ORF 7b as shown in Figure 1. (B) Plaque assay using crystal violet stain and immunofocus assay using a polyclonal protein patient serum reacting predominantly against the N protein (anti-N). (C) Relative Log RNA concentration (copies per mL) in viral supernatants after growth in cell lines as indicated. The zero value on the y-axis represents the starting RNA concentrations after virus absorption (1 h) and change of medium in each culture. Other data for each culture were normalized by subtraction of the logarithmic starting concentration. Each datum point shows the mean value of three independent experiments.

The recombinant virus containing the full-length ORF 7b gene was named rSCV. The virus containing the deletion in ORF 7b was termed r7bΔTMD. Both viruses were amplified on Vero cells and stored for further experiments. To confirm the purity of virus preparations, two different RT-PCR assays were done. The first assay utilized primers spanning the deletion in ORF 7b, as shown in Figure 5A. Both preparations yielded singular PCR products whose molecular weight was lower for r7bΔTMD than for rSCV. The molecular weight difference corresponded to the size of the ORF 7b deletion. For confirmation, a second RT-PCR assay was done with a primer hybridizing with the deleted portion of ORF 7b that was missing in r7bΔTMD. A singular band was obtained for rSCV but not for r7bΔTMD (Figure 5A). Identity of all PCR products was confirmed by sequencing.

### The 7b protein is expressed in cells during SARS-CoV infection

**Figure 6:**
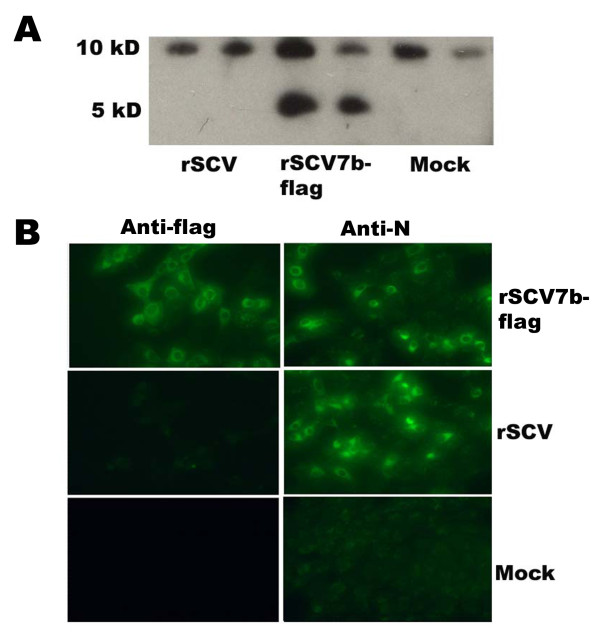
**Expression of ORF 7b**. (A) Detection of ORF 7b-flag expression with an anti-flag antibody by Western blot analysis. The 10 kD band is non-specifically detected in all samples. (B) Vero cells were infected with the flag-tagged recombinant virus rSCV7bflag or with the recombinant virus rSCV and subjected to IFA at 24 h p.i. IFA was done with anti-flag antibody (left panel, anti-flag) or a convalescent patient serum reacting predominantly against the SARS-CoV nucleocapsid protein (right panel, anti N).

Since an appropriate antibody directed against ORF 7b was not available when we started these studies, a DDDDK (flag-) tag sequence was introduced in the infectious clone prSCV by overlap-extension PCR at the C-terminus of ORF 7b. As shown in Figure 6A, a protein band corresponding to the predicted molecular weight of the 7b protein (5.3 kDa) was specifically detected in rSCV7bflag-infected cells using an anti-flag antibody. Also, immunofluorescence analyses revealed a dotted perinuclear pattern in rSCV7bflag-infected cells stained with an anti-flag antibody, whereas rSCV-infected cells incubated with the same antibody did not show fluorescence (Figure 6B). Expression of the nucleocapsid (N) protein was confirmed with a human serum directed mainly against N with both viruses (Figure 6B).

It was concluded that the ORF 7b protein of the recombinant viruses was expressed in infected cells, and that its principal properties are not affected by a C-terminal flag-tag epitope. These findings, including the pattern of fluorescence when expressing ORF 7b, were consistent with earlier reports by Pekosz *et al *[18,40].

### The deletion in ORF 7b enhances growth of virus in cell culture

Growth properties of rSCV and r7bΔTMD on different cell lines were compared. Plaque morphology was determined for both viruses, with no discernible differences (Figure 5B). Because plaque assay could only show cells that die from virus infection, the same experiment was repeated and read out by immunofocus assay, using serum of a human SARS survivor. There was no difference in immunofocus morphology (Figure 5B).

Growth curves in three different cell cultures were determined next. Virus RNA was measured in supernatant by real-time RT-PCR. A multiplicity of infection (MOI) of 0.001 was used for both recombinant viruses in Vero and CaCo-2 cells. For HuH7 cell, an MOI of 0.01 was used, due to their lower susceptibility to SARS-CoV infection. In Vero cells, very similar increases in RNA concentration were observed with both viruses during 48 hours (Figure 5C). In CaCo-2 and HuH7 cells, respectively, r7bΔTMD accumulated about 4- and 6-fold more RNA than rSCV. It was concluded that the deleted virus had a slight but reproducible growth advantage in the latter cell lines. In the absence of mechanisms of adaptive immunity, replication of RNA viruses is controlled by production of and response to type-I interferons, as well as apoptosis of infected cells. Taking into account our findings in overexpression experiments, central elements of these systems were therefore examined in cells infected with both virus variants.

### ORF 7b is not involved in the ablation of interferon induction observed during SARS-CoV infection

**Figure 7:**
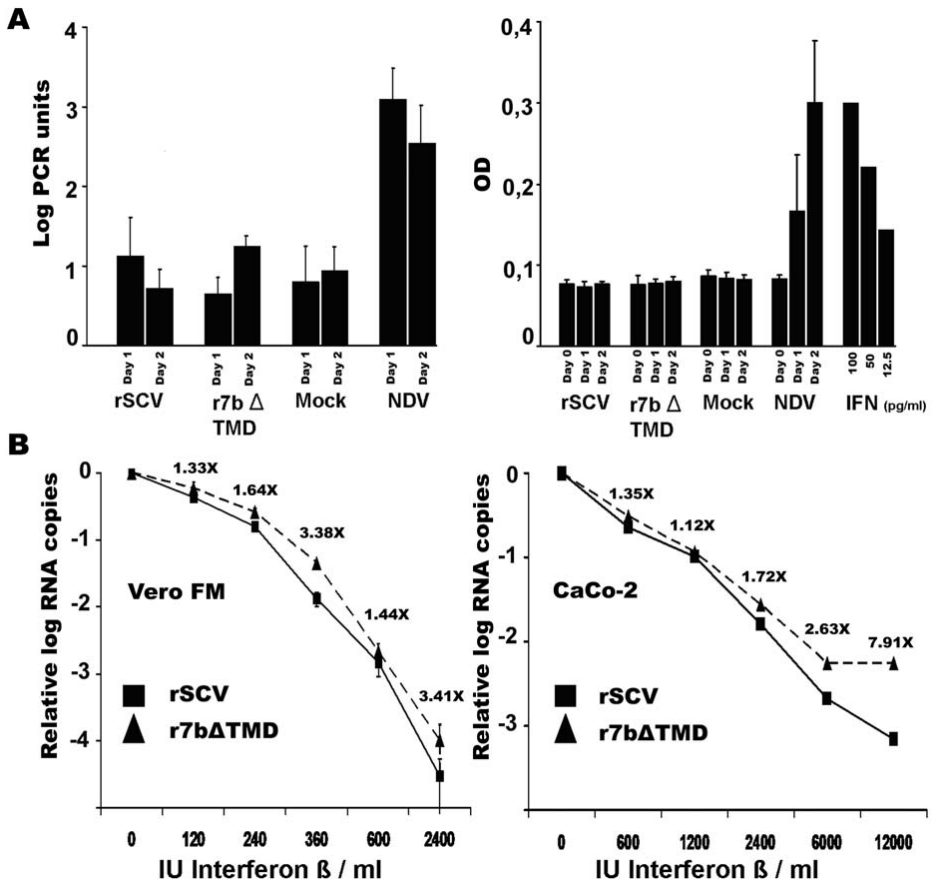
**Interferon induction, production and sensitivity**. (A) Left panel, interferon beta mRNA as quantified by real-time RT-PCR in 293-lp cells infected with rSCV or r7bΔTMD at an MOI of 5. Medium from mock-infected cells or cells infected at the same MOI with NDV served as controls. One PCR unit (y-axis) represents ten times the minimum concentration of interferon beta RNA detectable by the assay. (A) Right panel, interferon alpha secreted in supernatant of the same cells, as measured by EIA. The IFN standard exemplifies the sensitivity and linear range of the assay. (B) Viral RNA concentrations measured by real-time RT-PCR after two days of infection in cells pre-treated with increasing concentrations of interferon beta (x-axis). The left panel shows the results of triplicate experiments on Vero cells, the right panel shows results of duplicate experiments on CaCo-2 cells. For each graph the zero value indicates the Log RNA concentration achieved without interferon, to which the rest of the data were normalized. Viruses and cells used in each experiment are stated in the panels.

Because Vero cells as well as HuH-7 cells are deficient in interferon induction [50], HEK 293-lp cells were used to analyze interferon beta mRNA transcription. These cells have been shown to be capable of inducing and secreting interferon, and they are susceptible to SARS-CoV infection [50]. HEK 293-lp cells were seeded in six-well plates and infected with rSCV or r7bΔTMD at an MOI of 5. As shown in Figure 7A, infection with the control virus NDV elevated the transcription level of interferon beta mRNA by a factor of 100. rSCV did not induce interferon beta mRNA transcription, confirming earlier findings [50]. Induction of transcription was not observed with r7bΔTMD either, indicating that the ORF 7b protein is not involved in the ablation of interferon induction conferred during SARS-CoV replication. Essentially the same results were obtained with CaCo-2 cells (Figure 7B).

### ORF 7b does not interfere with interferon alpha production

HEK 293-lp cells were used to study release of interferon alpha in the supernatants of infected cells. It has been reported by Spiegel *et al*. that interferon alpha expression is induced in SARS-CoV-infected 293-lp cells to a certain level [50]. Exactly the same cells were obtained from F. Weber, University of Freiburg, and interferon alpha transcription after infection with SARS-CoV was qualitatively confirmed by RT-PCR in our laboratory (not shown). The level of interferon alpha was then determined by EIA in supernatant of 293-lp cells, 48 h after infection of both viruses at an MOI of 5. As shown in Figure 7A, infection with the control virus NDV elevated the interferon alpha level in supernatant by a factor of 3, while neither rSCV nor r7bΔTMD caused detectable secretion.

### Virus with the deletion in ORF7b has a slight replicative advantage in cells pretreated with interferon beta

To study the effects of interferon on replication of both viruses, Vero cells were pre-treated with increasing concentrations of interferon beta in order to induce an antiviral state. Cells were infected with either rSCV or r7bΔTMD at an MOI of 0.001. As shown in Figure 7B, r7bΔTMD replicated to marginally higher virus concentrations than rSCV in presence of interferon (up to 3.4 fold increase). Since in our hands CaCo-2 cells were more resistant to interferon beta pre-treatment than Vero cells, experiments were repeated with higher concentrations of interferon using CaCo-2 cells. More efficient replication (up to 7.9-fold increase) was again observed for r7bΔTMD (Figure 7B).

### The deletion in ORF 7b does not alter the capability of virus to induce apoptosis in cell culture

**Figure 8:**
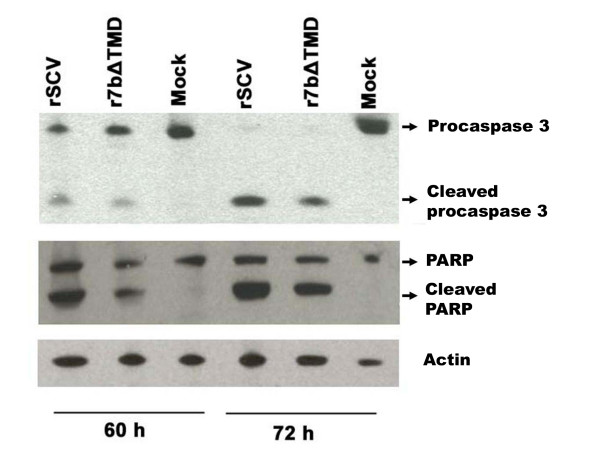
**Induction of apoptosis by recombinant coronaviruses rSCV and r7bΔTMD**. Vero FM cells were infected with rSCV or r7bΔTMD at an MOI of 5. Cleavage of caspase 3 and PARP-1 at 60 and 72 hours post infection was analyzed by Western Blot analysis.

Programmed, caspase-mediated death of infected cells is an efficient way of controlling virus replication. Several SARS-CoV accessory gene products have been implicated in the induction of apoptosis, including the ORF 7a and ORF 7b proteins as confirmed in this study (Figure 2). Activation of apoptosis was therefore compared in cells infected with either rSCV or r7bΔTMD. Vero cells were infected at an MOI of 5 of either virus and assayed by Western blot for activation of caspase 3, the central element of the apoptosis induction cascade. As opposed to the clear effect seen in overexpression experiments (Figure 2), both viruses induced partial cleavage of procaspase 3 at 60 hours post infection, and complete cleavage after 72 hours (Figure 8). To confirm these results we analyzed cleavage of poly-ADP ribose polymerase type 1 (PARP-1), a downstream effect of caspase-3 activation [51]. As shown in Figure 8, Western blot showed little differences in processing of PARP-1 in Vero cells with both viruses. It was concluded that the deletion-dependent ablation of the pro-apaptotic effect of ORF 7b as observed in overexpression experiments was irrelevant in the context of full virus replication in cell culture.

### The deletion in ORF 7b confers a significant replicative advantage in Syrian golden hamsters

**Figure 9:**
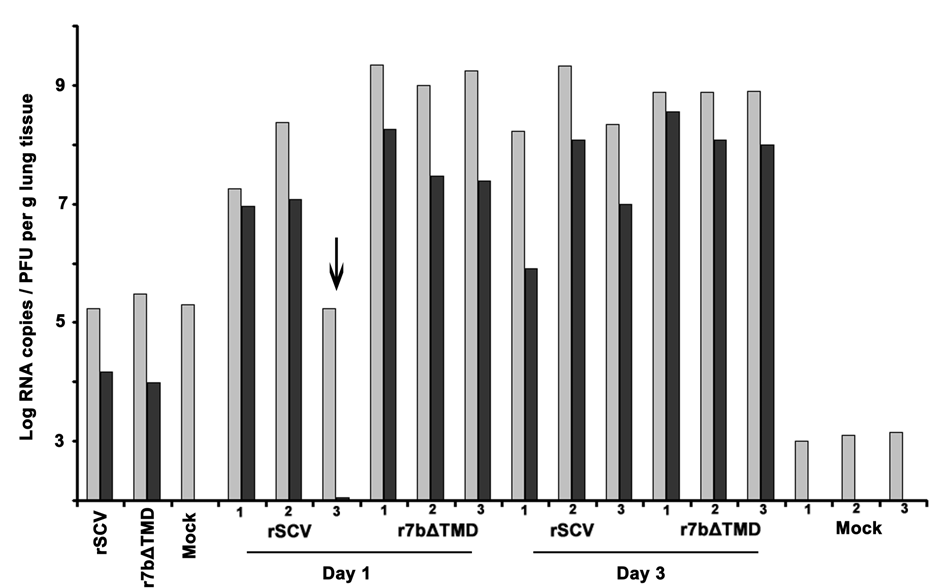
**In-vivo effect of the ORF7b deletion**. Golden Syrian hamsters were infected with 10^4 ^PFU of rSCV and r7bΔTMD (x-axis). Heat inactivated rSCV served as mock control. For each point of time post infection, three animals per virus variant were sacrificed (animals 1, 2, 3 as identified on the x-axis). Lungs were taken in total. Viral titers were determined by plaque assay and viral RNA was quantified by real-time RT-PCR. Light grey bars represent log copies of viral RNA, dark grey bars represent PFU per g lung tissue. The arrow indicates one animal with failure of virus replication.

Deletions in and around the sgRNA 7 region occurred during the 2003 epidemic and were transmitted in the community [25-27]. In order to elucidate whether the ORF 7b deletion might influence replication *in-vivo*, both viruses were tested in hamsters. Syrian Golden hamsters have been shown to be an acceptable rodent model for SARS-CoV replication and pathogenicity [52,53]. Four groups of three hamsters were infected via the intranasal route with 10^4 ^PFU of either rSCV or r7bΔTMD, and sacrificed on day 1 or 3, respectively. Whole lungs were minced and tested for infectious virus and viral RNA. The deleted virus yielded 95-fold more infectious particles and 23-fold more RNA copies in the lungs on day 1 (Figure 9 and Table 1). Differences decreased but remained qualitatively equivalent by day 3 (16-fold and 1.8-fold more infectious virus and RNA, respectively). The differences in RNA concentrations were borderline significant on day 1 (Table 1). T-tests did not identify further significant differences between our small groups of animals, and we did not want to use more animals for these experiments. In one of three animals sacrificed on day 1 post infection, rSCV failed to replicate entirely (Figure 9).

**Table 1:** Virus replication levels in hamster lungs

	Virus replication (mean* of N animals)		T-test*
	rSCV (Virus titer, RNA concentration, N animals)	r7bΔTMD (Virus titer, RNA concentration, N animals)	p
**Day 1 p.i.**	**1.04 × 10^7 ^**(9.00 × 10^6 ^- 1.2 × 10^7^) PFU/g	**9.86 × 10^8 ^**(2.4 × 10^7 ^- 1.8 × 10^8^) PFU/g	**0.15**
	**6.65 × 10^7 ^(**1.84 × 10^7 ^- 2.4 × 10^8^) copies/g	**1.53 × 10^9 ^**(9.98 × 10^8 ^- 2.2 × 10^9^) copies/g	**0.052**
	**n = 2**	**n = 3**	
			
**Day 3 p.i.**	**9.86 × 10^6 ^**(8.00 × 10^5 ^- 1.20 × 10^8^) PFU/g	**1.63 × 10^8 ^**(1.00 × 10^8 ^- 3.6 × 10^8^) PFU/g	**0.13**
	**4.27 × 10^8 ^**(1.68 × 10^8 ^- 2.1 × 10^9) ^copies/g	**7.70E × 10^8 ^**(7.50 × 10^8 ^- 8.10 × 10^8^) copies/g	**0.5**
	**n = 3**	**n = 3**	

* Means were determined upon logarithmic data and calculated back into linear values. Two-tailed t-tests were done on logarithmic values

The replication advantage for r7bΔTMD was in concordance with findings in CaCo-2 and HuH-7 cell cultures (Figure 5).

## Discussion

In the present study we have characterized a naturally-acquired deletion in the ORF 7b of the primary SARS-CoV Frankfurt-1 isolate by reverse genetics. In contrast to other plus strand RNA viruses it has taken rather long to complete the first coronavirus reverse genetics systems [28,30,31,34,37,54]. It has been difficult to clone complete CoV genomes due to their large sizes and toxicity or lability of constructs in *E. coli *[31,34,37]. This has been circumvented by Baric *et al*. by the use of subgenomic plasmids that are ligated *in-vitro *to full genomic cDNA, prior to transcription and electroporation [32]. We tried this approach initially, but we failed to generate sufficient amounts of full-length cDNA for in-vitro transcription. Thiel *et al*. have described an approach to generating full-length cDNA by stepwise assembly of an entire coronavirus genome in a pox virus backbone [34]. As we had not worked with pox viruses before, this technique appeared rather difficult to establish. As a third alternative, Enjuanes and coworkers have presented an approach based on cloning of the entire genome in BAC and transfecting the BAC-contained viral cDNA under the control of a CMV promoter [37]. The use of BAC DNA provides the remarkable benefit of being able to handle full length genomic DNA in one plasmid backbone, using standard DNA cloning techniques. As demonstrated in several studies of that group [24,36,37,55-57], BAC manipulations are rather fast and straightforward, while providing little opportunity for *de-novo *mutations resulting from DNA manipulation steps. In our strategy we used a bacteriophage T7-derived RNA polymerase promoter instead of the CMV promoter because we wanted to provide a genome that most closely resembled that of the virus, using cytoplasmic sites for replication and circumventing transcription and possible splicing in the nucleus [37,38,56]. A T7 promoter has not been used before with a plasmid-contained CoV cDNA genome; it was conceivable that leaky transcription might enhance underlying toxicity of CoV genomes in *E. coli*. Our study shows that the SARS-CoV genome is stable in BAC despite the T7 promoter. Interestingly, Enjuanes and colleagues have made BAC-based full length clones for different CoV and reported that their SARS-CoV BAC clone was more stable than, e.g., the one they developed for TGEV [36]. The SARS-CoV genome may thus be more stable in *E. coli *than that of other CoVs. It remains to be seen whether combined T7/BAC infectious cDNA clones can also be constructed for other CoVs.

The 45 nucleotide in-frame deletion in the transmembrane domain of ORF 7b is a paramount feature of the Frankfurt-1 strain. This strain has been employed as a prototypic SARS-CoV in several studies on pathogenesis and antiviral therapy (e.g., [42-45]). By analysis of primary clinical samples from the patients treated in 2003 for SARS in Frankfurt, we could show that the mutation has been selected during initial isolation in cell culture, and that it did not stem from the Frankfurt index patient [2]. Initial characterizations of the protein by overexpression experiments suggested reduced induction of interferon and apoptosis in association with the deletion, which led us to reconstruct the corresponding viruses with and without the deletion by reverse genetics. In concordance with earlier findings, type I interferon was neither induced nor produced by either SARS-CoV variant in our study [50,58-61]. It is assumed that CoV either encode a range of proteins interacting with interferon sensing, or shield their RNA from immune recognition through the formation of double membrane vesicle-based replication compartments [60,62-64]. Our experiments suggest that ORF 7b is not necessary for SARS-CoV counteraction against the induction of the interferon beta promoter. It also seems unlikely that ORF 7b contributes to the interference of SARS-CoV with secretion of interferon alpha [62]. However, the deleted virus showed slightly decreased sensitivity to pretreatment of cells with interferon. This effect was remarkable since earlier studies only determined opposite (= evasive) effects on the interferon response for CoV accessory proteins. These include interference with the interferon signalling cascade in the case of SARS-CoV protein 6, or prevention of activation of interferon-sensitive genes for mouse hepatitis virus nucleocapsid protein [61,65]. Here we observed an ORF 7b-dependent extension of the replication-attenuating effect of interferon. However, the additional extent of attenuation on top of the effect of interferon beta was of the same size as that observed in untreated cell cultures (compare Figure 5 and Figure 7) and did hardly increase with increasing interferon concentrations. This suggests an additive rather than a synergistic effect of ORF 7b and interferon on the attenuation of virus replication. In spite of the high relevance of the interferon response for controlling SARS-CoV replication, we should therefore assume that ORF 7b plays no role in the context of the type I interferon system [62,66].

Apoptosis of target cells can limit virus infection *in-vitro *and *in-vivo*. Our initial overexpression experiments pointed to a strong pro-apoptotic effect of intact ORF 7b, which was in concordance with a study by Schaecher *et al*. who found that sgRNA 7-derived proteins activated caspase 3 if overexpressed [40]. Complementary to their study, however, our experiments did not confirm any similar effect specifically for the ORF 7b protein in the full virus context. Schaecher *et al*. studied a recombinant virus with a double deletion of both ORF 7a and 7b, and this virus induced apoptosis clearly less efficiently than the parent full-length virus [18,19]. The most likely explanation for the difference between both viruses is that the pro-apoptotic effect of gene 7 proteins observed by Schaecher *et al*. was contributed by ORF 7a rather than ORF 7b.

Even though the ORF 7b deletion in Frankfurt-1 was not affecting interferon and apoptosis systems, the virus with a deletion seems to have been selected during isolation in cell culture and shows a replicative advantage in two of three cell lines. This is remarkable because SARS-CoV variants with deletions in the ORF 7 (and also ORF 8) gene region have been transmitted and maintained in humans in the late phases of the 2003 epidemic [25-27]. It has never been formally addressed whether these viruses might have undergone particularly efficient transmission. We therefore determined whether the ORF 7b deletion in Frankfurt-1 conferred a replicative advantage *in-vivo*, using Syrian Golden Hamsters as a model of human SARS-CoV infection [52,53]. Interestingly, the enhancing effect of the ORF 7b deletion was even more pronounced in hamsters than in cell culture. Hamsters infected with the deleted variant had significantly more virus RNA and a 95-fold increase of infectious virus titers in their lungs after 24 h. The rate of successful infections was 6/6 with the deleted virus and 5/6 with the full virus. In concordance with these observations, Roberts *et al*. have described ca. 10-fold more efficient replication of Frankfurt-1 in hamsters as compared to Urbani and HKU-39849 [52,53]. Mortality in Hamsters was only observed with Frankfurt-1 (3 of 20 animals) but not Urbani and HKU-39849 [52,53]. It was suggested that an amino acid exchange (L1148F) in the S2-domain of the spike protein of Frankfurt-1 against both Urbani and HKU-39849 might explain the difference. However, a replicative difference in extent similar to that reported by Roberts *et al*. was observed in our study between two variants of Frankfurt-1 that differed only by the ORF 7b deletion. As the deletion is not present in Urbani or any other prototype strain, this identifies the 7b protein as a potential attenuating factor within the genome of SARS-CoV.

We have seen in this study that the attenuating effect of ORF 7b was focused on the early phase of infection *in-vivo*. Because it has been suggested that delayed accumulation of high virus concentrations in infected patients has limited the spread of SARS-CoV in the population, it is tempting to speculate that the occurrence of viruses with deletions in the ORF7/8 region in the late phase of the 2003 epidemic might have added to the efficiency of virus transmission in humans [67-69]. It will be interesting in the future to investigate the exact mechanism of ORF 7b-dependent attenuation, and to determine whether this might contribute to the maintenance of virus in its natural reservoir.

## Materials and methods

### Cells and viruses

The original Vero cells on which Frankfurt-1 was primarily isolated (hereafter termed Vero FM, obtained from Jindrich Cinatl, Universtiy of Frankfurt), human hepatoma cells HuH7 (ATCC CCL-185), human colonic cancer cells CaCo-2 (ATCC HTB-37) and human embryonic kidney cells HEK 293-low passage (hereafter termed 293-lp, obtained from Friedemann Weber, University of Freiburg [50]) were maintained and grown in Dulbecco's modified Eagle medium (DMEM) containing 10% foetal calf serum (FCS, PAA, Pasching, Austria), 1 mM glutamine (PAA), 1 mM sodium pyruvate (PAA), 1% non-essential amino acids (PAA), 100 U/ml penicillin (PAA), and 100 μg/ml streptomycin (PAA). All experiments with 293-lp cells were performed between cell passage 42 and 48.

The SARS-CoV Frankfurt-1 isolate [2,20,70] was titrated on Vero FM cells. Sindbis virus (SV) derived from infectious cDNA clone pTOTO [28] was obtained from Beate Kümmerer, BNI, Hamburg and titrated on Vero FM cells. Sendai virus (SeV) strain Cantell was obtained from Christopher Basler, Mount Sinai School of Medicine, New York, propagated in 11-day-old embryonated chicken eggs, and titrated by standard hemagglutination test. Newcastle disease virus (NDV) strain PPMV-1/pigeon/Germany/R151 was obtained from the virus collection of the Friedrich Löffler Institute, Riems, Germany, and titrated on Vero FM cells.

### Virus quantification by cell culture and RT-PCR

Plaque assays were done with Avicel overlays (RC581, FMC BioPolymer, Belgium) as described elsewhere [71]. Immunofocus assay used the same overlay and was otherwise performed as described previously [72]. Viral RNA quantification using *in-vitro *transcribed RNA standards was done as described previously [2].

### General cloning and mutagenesis techniques

Standard cloning techniques were used. All gel purifications were done with the QIAEX II kit (Qiagen, Hilden, Germany). DNA constructs were electroporated into E. cloni (Lucigen, Middleton, USA) or Stbl 3 *E. coli *cells (Invitrogen, Karlsruhe, Germany). Prior to digestion with methylation-sensitive endonucleases plasmids were transformed in Sure cells (Stratagene, La Jolla, USA). BAC preparations were done with the Nucleobond^®^AX-kit (Macherey Nagel, Germany) as instructed. Plasmid-based inverse PCR was performed with QuikChange XXL kit (Stratagene, USA). PCR mutagenesis by overlap-extension PCR used Phusion^® ^DNA polymerase and around 50 ng of input plasmid DNA. SARS-CoV coding sequence within constructs was fully sequenced after every mutagenic step.

### Cloning of subgenomic plasmids

Total RNA was extracted from infected Vero cells with the Qiagen RNeasy kit. Using primers described by Yount *et al*. [32], cDNA fragments spanning the SARS-CoV genome were generated by RT-PCR using Superscript III reverse transcriptase and Expand High Fidelity DNA polymerase mixture. These primers inserted Bgl I restriction sites at fragment borders and a T7 promoter in front of the 5'end of the genome [32]. In addition to the strategy described by Yount *et al*., a Not I restriction site was introduced downstream of the genomic poly-A tail. Figure 3 gives an overview of cloned fragments. Fragment A was cloned in two parts (A1 and A2, Figure 3), using primer Afwd *5'-TACTAATACGACTCACTATAGATATTAGGTTTTTACCTACCCAGG-3' *and A1rev 5'-aatgccagtatgacctgagccaatatc-3' and A2fwd *5'-GATATTGGCTCAGGTCATACTGGCATT-3' *and Arev *5'-ACACCATAGTCAACGATGCC-3'*. After correction of errors both inserts were amplified from plasmids and used as templates in an overlap-extension PCR. A naturally existing Bgl I restriction site at genome position 1572 was thereby deleted. The extension product was subcloned in pSMART, resulting in clone pA. PCR products B, D, and E were cloned in pCR2.1 (Invitrogen). Fragments C and F were cloned in pSMART Low Copy Kanamycin vectors (Lucigen) after instability was observed in pCR2.1. A 45 nt deletion present in the Frankfurt-1 virus isolate (nt 27654 to 27699 in Genbank Accession No AY310120), was filled in by overlap-extension PCR. A region including restriction sites BamH I (genome position 26045) and Not I (following the 3'end of genome) was amplified from subclone pF in two halves using appropriate outer primers and overlap-extension primers *5'-TTTCTGCTATTCCTTGTTTTAATAATGCTTATTATATTTTGGTTTTCACTCGAAATCCAGGATCTAGAAG-3' *and *5'-ATTATTAAAACAAGGAATAGCAGAAAGGCTAAAAAGCACAAATAGAAGTCAATTAAAGTGAGCTCATTC-3'*.

The fragments were overlap-extended, digested with BamH I and Not I, and cloned back into the corresponding restriction sites in clone pF. All clones were verified by sequencing. Using the same technique, a DDDDK (flag-) tag sequence was introduced at the C-terminus of ORF 7b, with overlap-extension primers 5'-*GATTACAAGGATGACGACGATAAGTAAACGAACATGAAACTTCTC*-3' and 5'-*CTTATCGTCGTCATCCTTGTAATCGACTTTGGTACAAGGTTCT*-3'.

### Assembly of full length BAC cDNA clone

BAC vector pBeloBAC11 was obtained from NEB, Boston, USA. The Nco I site at position 890 was oblated by primer extension mutagenesis, resulting in pBelodNco. The Not I-Not I multiple cloning site fragment was removed from pBelodNco and replaced by an oligonucleotide adapter containing Nsi I, BsaH I, Sph I and Not I restriction sites in sequence, resulting in pBeloAd4. Fragment A was amplified from plasmid pA with primers 5'-*AGTAATGGGCCCTAAGTACTAATACGACTCACTATAGATATTAGG*-3' and *5'-ACACCATAGTCAACGATGCC-3'*, thereby introducing a PspOM I site upstream of the T7-promotor (Figure 3). The fragment was digested with PspOM I and Bgl I and ligated to the long EcoR I - Not I fragment of pBeloAd4 (pBeloAd4A, Figure 1). The 5'-most 3,062 nt were amplified from plasmid pB using primers *5'-GCCTATATGCATGGATGTTAGAG-3*' and 5'-*ATGAATGCGGCCGCTACACTCAACACGTGTGGCACGC*-3', thereby introducing a Not I site immediately downstream of the Mlu I site at position 7453. The PCR product was digested with Bgl I and Not I, gel purified, and ligated to the dephosphorylated short Not I - EcoR I fragment of pBeloAd4 (pBeloAd4B1, Figure 3). pBeloAd4A and pBeloAd4B were gel purified and ligated, resulting in quarter clone pAB, (Figure 3).

The 3'-most 5,536 nt were amplified from plasmid B3 in two parts, using primers *5'-TAGACTACGCCGGCGTAGCCTTAGGTTTAAAAACAATTGCCACTC-3' *and *5'-TACACTCAACACGTGTGGCACGATTGCGCT-3' *(5'-part); and primers *5'-AGCGCAATCGTGCCACACGTGTTGAGTGTA-3*'and *5'-TGAACCGCCACGCTGGCTAAACC-3' *(3'-part), respectively. Both products were overlap-extended, resulting in a PCR product with a depleted Mlu I site at position 7453. The product also contained a Not I site upstream of the Bsu36 I site at position 6544, introduced by a primer 5'-overhang. The product was Not I and Bgl I digested and ligated to the long EcoR I - Not I fragment of pBeloAd4 (pBeloAd4B2, Figure 3).

Plasmid pD was digested with Bgl I and Bcl I (compatible to BamH I). The fragment was ligated to the dephosphorylated short BamH I - EcoR I fragment of pBelodNco (pBeloNcoD1, Figure 3). Fragment C was cut out of its pSMART vector with Bgl I and dephosphorylated, followed by ligation to pBeloNcoD1 and gel purification. This product was ligated to pBeloAd4B2, generating quarter clone pBCD.

The Acl I - Bgl I fragment of vector D-24-5 was ligated to the long EcoR I - BsaH I fragment of pBelodNco (Bsa HI is compatible with Acl I) (pBeloNcoD2, Figure 3). The dephosphorylated Pst I - Bgl I fragment of vector pE was ligated to the short Nsi I - EcoR I fragment of pBeloAd4 (Nsi I is compatible with Pst I) (pBeloAd4E1, Figure 3). This product was ligated with pBeloNcoD2 to yield quarter clone pDE.

The 2,793 bp SpH I - Bgl I fragment of subclone pE was ligated to the long EcoR I - SpH I fragment of pBeloAd4 (pBeloAd4E2, Figure 3).

The Bgl I - Not I fragment of plasmid pF was ligated to the short Not I - EcoR I fragment of pBeloAd4 (pBeloAd4F, Figure 1). This fragment was ligated with pBeloAd4E2, yielding quarter clone pEF.

Quarter clones pAB and pBCD were digested with Bsu36 I and PspOM I, the latter cut destroying the replicative element *sopC*. Fragments of interest were gel-purified and ligated to yield half clone pL. Quarter clones pDE and pEF were digested with Nco I. One Nco I cut was in the virus cDNA insert on each BAC, and the other in the *sopC *gene. Fragments of interest were purified and ligated to yield half clone pR. Half clones were digested with Mlu I and PspOM I. Fragments of interest were gel purified and ligated into the full length clone prSCV.

### Rescue of recombinant virus

Full-length BAC clones were linearized with Not I, extracted with phenol-chloroform, and transcribed with the mMessage-mMachine^® ^T7 (Ambion, USA) at an input of 1 μg of DNA per 20 μl reaction. A PCR product spanning the nucleocapsid reading frame and the genomic 3'-prime end was generated with primers *N-fwd *(5'-*GGCCATTTAGGTGACACTATAGATGTCTGATAATGGACCCCAATC*), the underlined sequence representing an SP6 promoter) and *Frev (5'-TTTTTTTTTTTTTTTTTTTTGTCATTCTCCTAAGAAGC*-3'*)*. The product was purified and transcribed with mMessage-mMachine SP6 kit. Transcripts from both *in-vitro *transcription reactions were quantified photometrically. Genomic transcripts and N transcripts were co-electroporated at a 10:1 ratio into 10^7 ^BHK-21 cells, using a GenePulser instrument (Biorad, Germany) with two pulses of 1.5 kV, 25 μF and maximal resistance. Cells were left at room temperature for 10 minutes and seeded in 75 cm^2 ^flasks. In a biosafety-4 laboratory, electroporated BHK-21 cells were incubated at 37°C for 24 hours. Supernatants were serially diluted and transferred to Vero cells. Using 1% SeaPlaque^® ^agarose overlay (Biozym, Germany), three rounds of plaque purification were performed for each recombinant virus.

### Analysis of mutations in ORF 7b

To distinguish between the two genotypes, two different RT-PCRs were performed. RT-PCR 1 used primers 27500 fwd (5'-*CAGCTGCGTGCAAGATCAGT*-3') and 27900 rev (5'-*CCCTAGTGTTGTACCTTACAAG*-3'), thus comprising ORF 7b and yielding a 400 bp fragment for rSCV, while giving a 355 bp fragment for r7bΔTMD. For RT-PCR 2 the identical reverse primer was used but the binding site of the sense primer 27690 fwd (5'-*TAGCCTTTCTGCTATTCCTTGT*-3') was placed in ORF 7b, recognising the 45 nt only present in rSCV but deleted in r7bΔTMD, hence a PCR product was only obtained for rSCV.

### Cloning of ORF 7a, 7b and 7b del

ORF 7a (nts 27258 to 27626 of SARS-CoV genome GenBank accession number AY310120) was amplified using primers 5'-*CACCATGAAAATTATTCTCTTCCTGACA*-3' (fwd) and 5'-*TCATTCTGTCTTTCTCTTAATGGT*-3' (rev), and cloned into pCDNA 3.1. Because of low expression rates of the protein (data not shown) the insert was cloned into the high level expression vector pCAGGS [73], using *KpnI *and *NotI*. ORF 7b gene (nts 27623 to 27751) and the deletion mutant ORF 7b del gene (nts 27623 - 27751 deletion of 45 nts 32-76) were amplified using primers 5'-*CTAGAATTCCTCGAGACAATGAGAAGTTTCATGTTC*-3' and 5'-*ATCGTCGACCTCGAGTCACCATTAAGAGAAAGACAG*-3', and cloned into pI.18 vector (kind gift of Jim Robertson, National Institute for Biological Standards and Control, Hertfordshire, UK) with a T7 promoter that was inserted by standard cloning procedures. Expression of constructs was verified by coupled *in-vitro *transcription and translation using the TNT T7 Coupled Reticulocyte Lysate System (Promega, Mannheim, Germany) and immunofluorescence analysis of transfected cells (data not shown).

### ISG-54 reporter gene assay

Transfection of 293 cells was performed using the calciumphosphate transfection kit (Invitrogen) according to the manufacturer's instructions. 10^6 ^cells were transfected with 0.3 μg of the interferon (IFN)-stimulated response element (ISRE)-driven firefly luciferase reporter plasmid pHISG-54-Luc (kind gift of D. Levy, New York University School of Medicine, New York), 0.3 μg of the constitutive *Renilla *luciferase expression plasmid pRL-SV40 (Promega) and 4 μg of the plasmid of interest. 16 μg of herring sperm DNA (Promega) were transfected along with the plasmids to optimize DNA uptake. 24 h post transfection, cells were infected with SeV (20 hemagglutinating units) to induce the type I IFN response or were not infected. At 24 h post infection (p.i.), cells were harvested and lysed in 100 μl of passive lysis buffer (Promega, Mannheim, Germany). Subsequent luciferase assays were performed by using the Promega DUAL luciferase assay system according to the manufacturer's instructions. Relative renilla luciferase production was used to normalize for transfection efficiency.

### Immunofluorescence microscopy

Cells were seeded on chamber slides (μSlide^® ^8 well, Ibidi, Martinsried, Germany) and infected with SARS-CoV t a multiplicity of infection of 1. After 24 hours cells were washed once with PBS and fixed in ice cold acetone for 15 minutes. Prior to antibody staining, cells were washed three times with PBS. Reconvalescent SARS patient serum from our own diagnostic laboratory was diluted 1:1000 in PBS-T. Rabbit polyclonal antibody against the DDDDK - tag (Abcam, UK) was diluted 1:10000 in PBS-T. Cells were overlaid with 100 μl of antibody solution, incubated at 37°C for 1 hour, and washed four times for 5 minutes with PBS containing 0.1% Tween 20 (PBS-T). Fluorescein-conjugated goat anti-human or anti-rabbit IgG serum (Calbiochem/VWR, Darmstadt, Germany) was diluted 1:10000 in PBS-T and incubated at 37°C for 30 minutes. Cells were washed 4 times with PBS. Chambers were overlaid with 200 μl of PBS and a few drops of mineral oil. Fluorescence was analysed on an inverted fluorescence microscope.

### Western blot analysis

Subconfluent 293-lp cells in six well-plates were infected, harvested at different time points after infection, and pelleted by centrifugation for 5 minutes at 1200 rpm. Pellets were dissolved in 50 μl of 1× Chaps buffer containing 1 mM PMSF and 5 mM DTT followed by three freeze/thaw cycles. Nuclei were pelleted by centrifugation for 10 minutes at 16,000 g and the clarified lysate was dissolved in 1× SDS loading buffer. 10 μl of postnuclear lysate were loaded on precast 4-12% Bis-Tris gradient gels. Separated proteins were electroblotted on nitrocellulose membranes (Whatman, Dassel, Germany) and blocked for 1 h with 1× RotiBlock (Roth, Karlsruhe, Germany). Membranes were washed with PBS and incubated over night with primary antibody at 4°C. Rabbit polyclonal anti-caspase-3 and anti-PARP antibodies (Cell Signaling, Danvers, USA) were diluted 1:1000 in PBS-T. Rabbit anti-flag antibody (Abcam, Cambridge, UK) was diluted 1:5000 in PBS-T. Membranes were washed 4 times for 5 minutes with PBS-T. Horseradish peroxidase-conjugated goat anti-rabbit antibody (Cell Signaling, Danvers, USA) was used at 1:2000 dilution in PBS-T and incubated on membranes for 1 hour. Membranes were washed four times with PBS-T before LumiGLO reagent (Cell Signaling, Danvers, USA) was added. Membranes were exposed to scientific imaging film (Sigma-Aldrich, Munich, Germany) for appropriate times before development.

In overexpression experiments, Vero E6 cells were transfected with Lipofectamine 2000 (Invitrogen) according to the manufacturer's instructions. 5 × 10^4 ^cells were transfected with 1 μg of empty pI.18 vector, pCAGGS-ORF 7a, pI.18-ORF 7b or pI.18-ORF 7b del, respectively. At 48 h post transfection, cells were lysed in 50 μl 1× Chaps buffer. Proteins were separated on 15% SDS polyacrylamide gels, transferred onto PVDF membranes and blocked for 1 h with 5% skim milk (w/v) in PBS-T. Membranes were washed with PBS-T and incubated over night with primary antibody at 4°C. Western blot detection was done with horseradish peroxidase-conjugated goat anti-rabbit secondary antibody using an enhanced chemiluminescence detection reagent kit (Pierce, Perbio Science, Bonn, Germany) according to the manufacturer's protocol. Immunoreactive bands were visualized using an Optimax 2010 imaging system (PROTEC processor Technology, Oberstenfeld, Germany) with high performance chemiluminescence films (GE Healthcare, Munich, Germany).

### IFN-α ELISA

Interferon alpha was detected with the Human IFN Alpha ELISA Kit (PBL Interferonsource, Piscataway, USA) according to the manufacturer's instructions. Briefly, 100 μl of supernatant of samples and controls were added to pre-coated microtiter plates and incubated at room temperature for 1 hour, followed by one washing step, addition of antibody solution and another hour of incubation. After three washing steps, 100 μl of HRP conjugate concentrate were added and incubated for 1 hour. The plate was washed four times and TMB substrate was added. After 15 minutes of incubation stop solution was added and absorbance was determined at 450 nm.

### Quantification of interferon beta mRNA

Total RNA was prepared from 293-lp cells in 6-well plates with Trizol^® ^reagent (Invitrogen, USA) according to the manufacturer's instructions. RNA was quantified photometrically and 150 ng per reaction were analysed by real-time RT-PCR. Interferon beta mRNA was amplified with primers *IFNβFwd *(5'-*GAACTTTGACATCCCTGAGGAGATT*-3') and *IFNβRev *(5'-*GGAGCATCTCATAGATGGTCAATG*-3'), and 5'-nuclease probe *IFNβ-P *(FAM-*CAGCAGTTCCAGAAGGAGGACGCC*-TAMRA). GAPDH mRNA was detected in parallel with primers *GAPDHFwd *(5'-*AGGTGGTCTCCTCTGACTTCAACA*-3'), *GAPDHRev *(5'-*AGTGGTCGTTGAGGGCAATG*-3'), and probe *GAPDH-P *(FAM-*CACCCACTCCTCCACCTTTGACGCT*-TAMRA). Reactions using the OneStep RT-PCR kit (Qiagen, Hilden, Germany) comprised 50°C for 30 minutes, followed by 95°C for 15 minutes and 40 cycles of 95°C for 10 seconds and 58°C for 30 seconds. For both genes standard curves were generated from limiting dilution series of quantified RNA. The dilution end-points were defined as one PCR unit for each gene. Log PCR units for each experimental sample were calculated from the linear equations of the dilution series. Interferon beta quantity was normalised to GAPDH quantity by subtraction of logarithmic quantities (Interferon - GAPDH).

### Hamster infections

Infections were performed with rSCV and r7bΔTMD. Heat-inactivated rSCV served as the mock-control. Syrian Golden hamsters (strain LVG, Charles River Laboratories) were infected via the intranasal route with 10^4 ^PFU each. 100 μl of virus solution were applied. Hamsters were sacrificed at indicated days post infection. Lungs were prepared in total, weighed, and homogenized. Tissue was suspended to a concentration of 0.5 g/ml in complete DMEM before analysis by RT-PCR or cell culture.

## Competing interests

The authors declare that they have no competing interests.

## Authors' contributions

SP constructed the infectious clone and conducted all experiments with recombinant viruses. VK and EM designed and carried out the overexpression experiments and the reporter assays and/or critically revised the manuscript. VD conducted hamster infections. KG participated in construction of the infectious clone. CD designed the study, participated in the construction of the infectious clone, and wrote the manuscript. All authors took part in manuscript preparation. All authors read and approved the final manuscript.
